# Supplementary material for: Translational regulation enhances distinction of cell types in the nervous system
Source: eLife. 2024 Jul 16;12:RP90713. doi: 10.7554/eLife.90713 (PMC11251722; doi:10.7554/eLife.90713)
Supplement: Supplementary file 3. [file elife-90713-supp3.docx]

Sequences of the biotinylated 2′-*O*-methyl oligonucleotides for rRNA-depletion.

5´-/biotin/AUUGGCAUCACAUCCAUUGUCGUUUA-3´

5´-/biotin/ACAUCCAUUGUCGUUUAUAAAGUAAA-3´

5´-/biotin/AUAAACUUUAAAUGGUUUAGAAGCCAU-3´

5´-/biotin/AAAUGGUUUAGAAGCCAUACAAUGCAAA-3´

5´-/biotin/GCCUCAUUUAAGAAGGACUUAAAUCGUUAA-3´

5´-/biotin/AACGCCCCGGGAUUGUGUUAAUUAGCUA-3´

5´-/biotin/ACGUUAUACGGGCCUGGCACCCUCUAUGGGU-3´

5´-/biotin/AUUGUUAAUCAUACAAGUGCAUAUAAU-3´

5´-/biotin/AUAAAUCUAUCAGCACUUUAUCAA-3´

5´-/biotin/UUUAAUCAAGUAAGUAAGGAAACA-3´

Sequence of the *Rh1-Venus* reporter

10xUAS-hsp70Bb_promoter-Rh1_5′ leader-Venus-Rh1_3′ UTR

TAAGCTGCAGGTCGGAGTACTGTCCTCCGAGCGGAGTACTGTCCTCCGAGCGGAGTACTGTCCTCCGAGCGGAGTACTGTCCTCCGAGCGGAGTACTGTCCTCCGAGCGGAGACTCTAGCGAGCACGCGTCTGCAGGTCGGAGTACTGTCCTCCGAGCGGAGTACTGTCCTCCGAGCGGAGTACTGTCCTCCGAGCGGAGTACTGTCCTCCGAGCGGAGTACTGTCCTCCGAGCGGAGACTCTAGCGAGCCATATGAGCGCCGGAGTATAAATAGAGGCGCTTCGTCTACGGAGCGACAATTCAATTCAAACAAGCAAAACATTGCAGGTTTCCAACGACCAATCGCCGCGACTAGTCCGCCCCAGTGAAATATTCAGAATCCAGGAACCCTTTATGTAAAAAGTGTTAGAAATATTGTTAGTGAATTTGCAGCTTTTTATGTAGACAGTGTGATATAGGCGGGATATAGTGACGCAGCCAGTAACCAAAACACAATGGAGAGCTTTGCAGGCGCGCCAGTGAGCAAGGGCGAGGAGCTGTTCACCGGGGTGGTGCCCATCCTGGTCGAGCTGGACGGCGACGTAAACGGCCACAAGTTCAGCGTGTCCGGCGAGGGCGAGGGCGATGCCACCTACGGCAAGCTGACCCTGAAGCTCATCTGCACCACCGGCAAGCTGCCCGTGCCCTGGCCCACCCTCGTGACCACCCTGGGCTACGGCCTGCAGTGCTTCGCCCGCTACCCCGACCACATGAAGCAGCACGACTTCTTCAAGTCCGCCATGCCCGAAGGCTACGTCCAGGAGCGCACCATCTTCTTCAAGGACGACGGCAACTACAAGACCCGCGCCGAGGTGAAGTTCGAGGGCGACACCCTGGTGAACCGCATCGAGCTGAAGGGCATCGACTTCAAGGAGGACGGCAACATCCTGGGGCACAAGCTGGAGTACAACTACAACAGCCACAACGTCTATATCACCGCCGACAAGCAGAAGAACGGCATCAAGGCCAACTTCAAGATCCGCCACAACATCGAGGACGGCGGCGTGCAGCTCGCCGACCACTACCAGCAGAACACCCCCATCGGCGACGGCCCCGTGCTGCTGCCCGACAACCACTACCTGAGCTACCAGTCCGCCCTGAGCAAAGACCCCAACGAGAAGCGCGATCACATGGTCCTGCTGGAGTTCGTGACCGCCGCCGGGATCACTCTCGGCATGGACGAGCTGTACAAGTAAGCGGCCGCATTCTTTGGCGCAACAACCAGAACAGCAACAACAACAACAAGAACATCTAACTACTTACAACAGCAACAACAACAGCAACAAAAACAACAGCAAGAACAACTGCAGCAACAGAACGAAACGCTTTCGAATAACATCAAAAACTTCAACAATAATGAAAAAATTATGCAACTTTCTTACATAACAAAAAGCAATGTAAACTCAGTTATTAAATTTCCTGCAATGTCAGTTAAGGACAAAAAAAAACTCAACAAAAAAAATAAATGCAAACGAACTAGAAAAGTTATAAATTAAAATGAGCCTTTTCAAAACATAGTATATCTAACAAAAGCAGCTTTTAGCGTGGAAAAACCCTAATGACGAACCTACAAAAGTTCGGATATCAACTTTCGGTTATCTTTCGCCTTTAAAGTTTGGAGAACCACAACAAATTTGAGTTTATTCATTCTTATATGTATAATAGTCTTCTTCAGAAGCTATAAATCCTTTCCAGGCATGCACTTGGCTT

Sequence of the mutated *Rh1-Venus* reporter

10xUAS-hsp70Bb_promoter-Rh1_m_5′ leader-Venus-Rh1_3′ UTR

TAAGCTGCAGGTCGGAGTACTGTCCTCCGAGCGGAGTACTGTCCTCCGAGCGGAGTACTGTCCTCCGAGCGGAGTACTGTCCTCCGAGCGGAGTACTGTCCTCCGAGCGGAGACTCTAGCGAGCACGCGTCTGCAGGTCGGAGTACTGTCCTCCGAGCGGAGTACTGTCCTCCGAGCGGAGTACTGTCCTCCGAGCGGAGTACTGTCCTCCGAGCGGAGTACTGTCCTCCGAGCGGAGACTCTAGCGAGCCATATGAGCGCCGGAGTATAAATAGAGGCGCTTCGTCTACGGAGCGACAATTCAATTCAAACAAGCAAAACATTGCAGGTTTCCAACGACCAATCGCCGCGACTAGTCCGCCCCAGTGAAATATTCAGAATCCAGGAACCCTTTCCCAAAAAAGTGTTAGAAATATTGTTAGTGAATTTGCAGCTTTTTCCCAAAACAGTGTGATATAGGCGGGCCCAAACGACGCAGCCAGTAACCAAAACACAATGGAGAGCTTTGCAGGCGCGCCAGTGAGCAAGGGCGAGGAGCTGTTCACCGGGGTGGTGCCCATCCTGGTCGAGCTGGACGGCGACGTAAACGGCCACAAGTTCAGCGTGTCCGGCGAGGGCGAGGGCGATGCCACCTACGGCAAGCTGACCCTGAAGCTCATCTGCACCACCGGCAAGCTGCCCGTGCCCTGGCCCACCCTCGTGACCACCCTGGGCTACGGCCTGCAGTGCTTCGCCCGCTACCCCGACCACATGAAGCAGCACGACTTCTTCAAGTCCGCCATGCCCGAAGGCTACGTCCAGGAGCGCACCATCTTCTTCAAGGACGACGGCAACTACAAGACCCGCGCCGAGGTGAAGTTCGAGGGCGACACCCTGGTGAACCGCATCGAGCTGAAGGGCATCGACTTCAAGGAGGACGGCAACATCCTGGGGCACAAGCTGGAGTACAACTACAACAGCCACAACGTCTATATCACCGCCGACAAGCAGAAGAACGGCATCAAGGCCAACTTCAAGATCCGCCACAACATCGAGGACGGCGGCGTGCAGCTCGCCGACCACTACCAGCAGAACACCCCCATCGGCGACGGCCCCGTGCTGCTGCCCGACAACCACTACCTGAGCTACCAGTCCGCCCTGAGCAAAGACCCCAACGAGAAGCGCGATCACATGGTCCTGCTGGAGTTCGTGACCGCCGCCGGGATCACTCTCGGCATGGACGAGCTGTACAAGTAAGCGGCCGCATTCTTTGGCGCAACAACCAGAACAGCAACAACAACAACAAGAACATCTAACTACTTACAACAGCAACAACAACAGCAACAAAAACAACAGCAAGAACAACTGCAGCAACAGAACGAAACGCTTTCGAATAACATCAAAAACTTCAACAATAATGAAAAAATTATGCAACTTTCTTACATAACAAAAAGCAATGTAAACTCAGTTATTAAATTTCCTGCAATGTCAGTTAAGGACAAAAAAAAACTCAACAAAAAAAATAAATGCAAACGAACTAGAAAAGTTATAAATTAAAATGAGCCTTTTCAAAACATAGTATATCTAACAAAAGCAGCTTTTAGCGTGGAAAAACCCTAATGACGAACCTACAAAAGTTCGGATATCAACTTTCGGTTATCTTTCGCCTTTAAAGTTTGGAGAACCACAACAAATTTGAGTTTATTCATTCTTATATGTATAATAGTCTTCTTCAGAAGCTATAAATCCTTTCCAGGCATGCACTTGGCTT

Sequences of the *Venus* or *GFP* probes (18 nt, 31 probes)

#1:CGGTGAACAGCTCCTCGC, #2:GACCAGGATGGGCACCAC, #3:GTTTACGTCGCCGTCCAG, #4:CCGGACACGCTGAACTTG, #5:TTGCCGTAGGTGGCATCG, #6:GTGGTGCAGATGAGCTTC, #7:AGGGTGGTCACGAGGGTG, #8: AAGCACTGCAGGCCGTAG, #9:ATGTGGTCGGGGTAGCGG, #10:TGAAGAAGTCGTGCTGCT, #11:ACGTAGCCTTCGGGCATG, #12:AGAAGATGGTGCGCTCCT, #13:TCTTGTAGTTGCCGTCGT, #14:TCGAACTTCACCTCGGCG, #15:TCGATGCGGTTCACCAGG, #16:TGAAGTCGATGCCCTTCA, #17:CAGGATGTTGCCGTCCTC, #18:TTGTACTCCAGCTTGTGC, #19:AGACGTTGTGGCTGTTGT, #20:TTCTTCTGCTTGTCGGCG, #21:TGAAGTTGGCCTTGATGC, #22:CTCGATGTTGTGGCGGAT, #23:TAGTGGTCGGCGAGCTGC, #24:CGATGGGGGTGTTCTGCT, #25:TTGTCGGGCAGCAGCACG, #26:GGACTGGTAGCTCAGGTA, #27:GTTGGGGTCTTTGCTCAG, #28:ACCATGTGATCGCGCTTC, #29:CGGTCACGAACTCCAGCA, #30:TCCATGCCGAGAGTGATC, #31:CCGCTTACTTGTACAGCT
